# Colesional Cutaneous Kaposi Sarcoma and Cryptococcosis

**DOI:** 10.4269/ajtmh.20-1097

**Published:** 2021-02

**Authors:** Abdullah Ismail, Silindile Sibisi, Sugeshnee Pather

**Affiliations:** 1 Anatomical Pathology, National Health Laboratory Service, Chris Hani Baragwanath Academic Hospital, Faculty of Health Sciences, University of the Witwatersrand, Johannesburg, South Africa;; 2 Department of Dermatology, Gauteng Department of Health, Chris Hani Baragwanath Academic Hospital, Faculty of Health Sciences, University of the Witwatersrand, Johannesburg, South Africa

Herein, we present a rare case of colesional cutaneous acquired immunodeficiency syndrome-related Kaposi sarcoma (KS) and cryptococcosis in an adult male. Histopathological examination of persistent or progressive cutaneous lesions, occurring in the context of human immunodeficiency (HIV) infection, is likely to detect potentially life-threatening opportunistic infection (OIs) and/or neoplasia.

**Figure 1.**
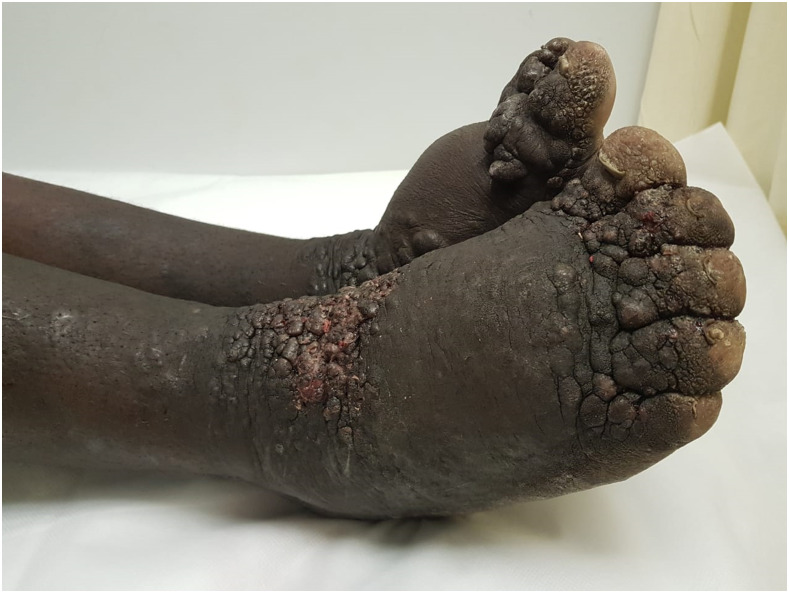
Lymphedema, violaceous nodules, and warty appearance of the lower limbs. This figure appears in color at www.ajtmh.org.

A 38-year-old man presented at the largest hospital in Africa with 6 years’ history of progressive lower limb swelling and cutaneous lesions. The patient was seropositive HIV and antiretroviral therapy naive. Physical examination confirmed his generally unwell status due to emaciation, generalized lymphadenopathy, and massive lymphedema of the lower limbs. Hyperpigmented plaques on the legs were intermixed with violaceous nodules. A prominent warty appearance and toenail destruction were evident (Figure 1). The clinical differential diagnosis included KS and acroangiodermatitis of Mali (pseudo-KS).

**Figure 2.**
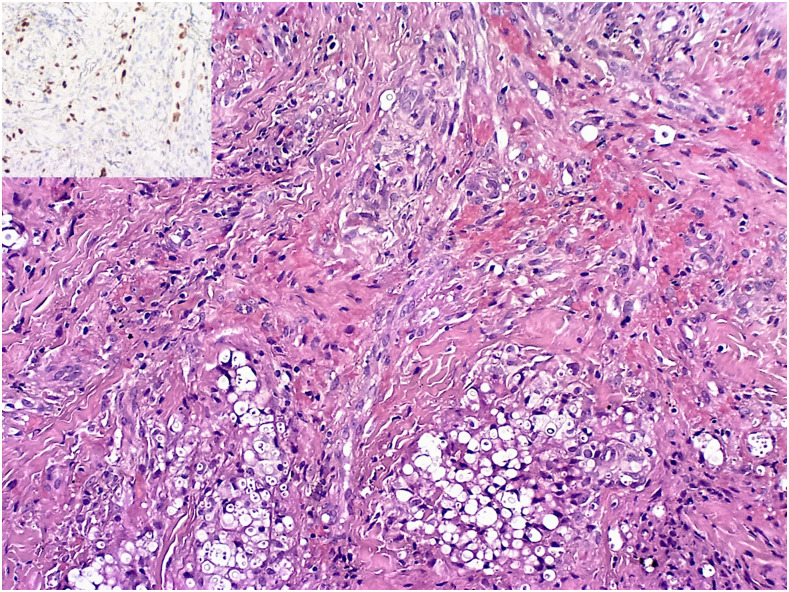
Colesional Kaposi sarcoma (inset displays HHV-8 immunoreactivity) and cryptococcosis (H&E stain at ×100 magnification). This figure appears in color at www.ajtmh.org.

**Figure 3.**
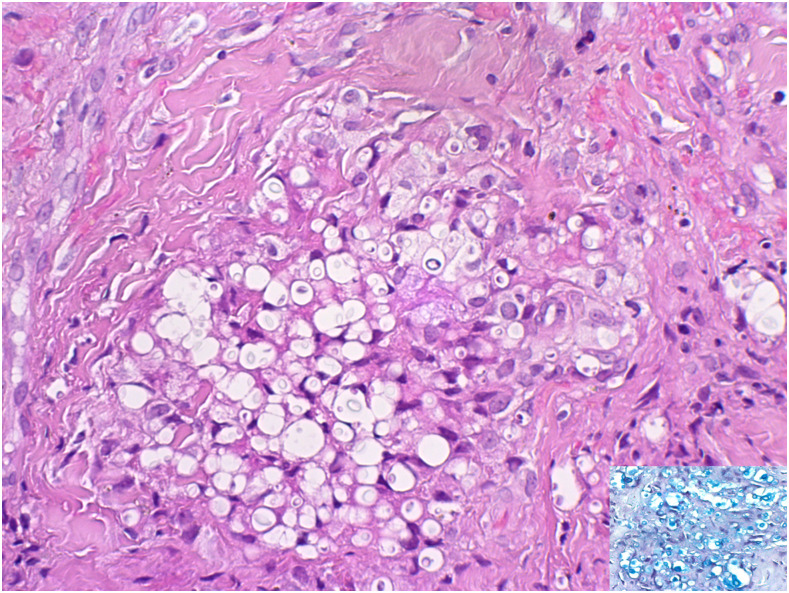
Colesional cryptococcosis (Alcian blue stain inset) and Kaposi sarcoma (H&E stain at ×200 magnification). This figure appears in color at www.ajtmh.org.

The HIV viral load was 4,75,000 copies/mL, the CD4 count was 5 cells/µL, and a positive serum cryptococcal latex agglutination test was confirmed. Histopathological examination of a skin lesion confirmed the presence of colesional KS and cryptococcosis (Figures 2 and 3).

Antiretroviral therapy and antifungal therapy were initiated. Unfortunately, death ensued while this patient was hospitalized.

Cutaneous manifestations of HIV may develop in more than 90% of infected individuals.^1,2^ However, the co-existence of KS and cryptococcosis within a cutaneous lesion is a very rare occurrence.^3–7^ Although antiretroviral therapy has led to a reduction in the incidence of KS and OIs, these continue to be the presenting conditions in the context of undiagnosed HIV infection. Contributing factors may also include suboptimal access to healthcare, nonadherence to antiviral therapy, or drug resistance. The presence of OIs is often an indicator of severe immunosuppression with disseminated disease. Therefore, the recognition of cutaneous involvement by OIs should expedite access to treatment.^8^

This report contributes to the expanding spectrum of colesional cutaneous pathology that manifests because of HIV-induced immunosuppression.
